# Extracts and Gleanings

**Published:** 1875-07

**Authors:** 


### Extracts and Gleanings.

*Indications for Thoracentesis.*—In a communication on the subject of pleuritic effusion (*British Medical Journal*), Dr. J. R. Wardell, of Tunbridge Wells, thus states the conditions which may be regarded as the morbid states, and the positive and negative signs, demanding the operation:

1. In all cases in which inspection and the physical signs give evidence of a large quantity of fluid, when there are symptoms of compression of the lung, and there is manifest cardiac displacement.

2. When there are urgent dyspnœa, an irregular pulse, and threatening of orthopnœa.

3. When the affected side is smooth and rounded, and the intercostal spaces are effaced or protrude; when measurement proves bulging; when the dullness in the chest is complete, or demarcated, and absolute; when there is abolition of tactile fremitus; when there are broncho-phonic voice, tubular breathing, and absence of breath-sound; when the patient can only lie on one side, or in diagonal position; and when there is the Hippocratic sign of succussion.

4. When the exploratory needle proves the fluid to be purulent.

5. If the heart be pushed from its normal situation, and the apex be substernal or beyond the right sternal edge, or if it be thrust toward the left hypochondrium, or if it be lost; when it becomes presumptive that the organ has been driven inward and backward; and when on the one side the liver depends abnormally into the abdomen, and when on the other side the relaxed and down-pressed diaphragm so displaces the spleen that its free edge can be *felt.*

6. When half the thoracic cavity is filled, and a month or so shows no proof of absorption, the longer the delay the less are the chances of expansion.

7. In those exceptional cases of double pleurisy, when both cavities become half filled with effusion, and dyspnœa shows the lung-space to be dangerously encroached upon.]

8. In pulmonary phthisis, when the accumulation of serous or sero-purulent secretion causes distress, and when the other lung assumes the symptoms of bronchitis or pneumonia, the operation should at once be performed.

9. In mechanical hydrothorax it may be had recourse to, though with no object to cure, but merely with a view, for a time, to prolong life and to aid in the action of medicinal remedies.

10. In children, whose chest-walls are thin, and in whom the white tissues are more developed and confer greater resiliency to the thoracic parietes, and whenever there are certain evidences of fluid, it should without delay be evacuated.

11. In hydropneumothorax it may be generally with safety and benefit employed.

12. Pointing externally should never be waited for.

13. Under certain circumstances repeated tappings are required. —*N. Y. Med. Journal.*

*Diphtheria.*—It seems not to be generallv known that diphtheria is an exanthematous affection having its characteristic cutaneous eruption. The eruption is very frequently absent, and when present so nearly resembles the rash of scarlet fever, that those not familiar with the nice distinctions which mark the various cutaneous exanthemata are very apt to class the disease as scarlatina, roseola or measles. We recently met with a case of this kind—the first we had ever seen attended with a rash, and should have unhesitatingly diagnosed it as scarlatina had we not observed the diphtheritic deposits on the tonsils with the odor peculiar to that affection.

Dr. Ransome, in the *Medical Times and Gazette,* in commenting upon the resemblance and possible relation between scarlatina and diphtheria, draws the following points of distinction: "The points of unlikeness in the nature and symptoms of the two diseases are too great to allow them to rank as varieties of the same species; thus the rash of diphtheria is often absent, is very variable as to the time of its appearance, occurring seldom at the outset of the malady, and it may occur as late as the third week. Albuminuria is often found within two or three days of the commencement of diphtheria, whilst in scarlatina it seldom sets in until desquamation of the kidney has commenced. There is an absence of definiteness about the duration of symptoms in diphtheria which marks it off from the regular sequence of events in scarlet fever; in the former, exudation may form on the fauces for six weeks or two months, without much affection of the cervical glands. The mode in which diptheria localizes itself on the mucous membranes or on broken surfaces separated it from any other disease. Different epidemics differ widely in the point selected for attack; often the larynx and bronchi have been seized, as well as the throat. But does scarlet fever attack the larynx? It is remarkable that in epidemics affecting other mucous membranes, with little fatality, the subsequent complications were frequent and troublesome. Lastly, does an attack of diphtheria confer any immunity from subsequent seizures? The susceptibility of the throat seems rather increased than diminished, while scarlatina seldom reappears in the same individual. It is evident that the two diseases are distinct, and yet that some very definite and close connection exists between them. It is not easy to point out wherein the bond consists; but it is possible that the relations of some other ferments may throw some light on the subject. M. Bertholot's discovery of an unorganized glucosic ferment derived from yeast (*Comptes Rendus*, vol. I. p. 980,) and the fact that one fermentation by organized beings frequently prepares the way for a different one, suggest two ways in which the singular relation existing between scarlatina and diphtheria might be accounted for." R. C. W.

*Hydrochloral by the Rectum in the Vomiting of Pregnancy.*—Dr. Simmons, the chief surgeon to Ken Hospital, Yokohama, Japan, remarks that he does not remember having seen hydrochloral, by the rectum, recommended in the vomiting of pregnancy. Should this application of it, however, not be new, the results of the following observations may still have their value for or against the conclusions already arrived at. Aware of the suddenness with which this symptom sometimes ceases, after all hopes of saving the mother, without emptying the uterus, have failed, and that, too, without being able to attribute it to any of the numerous remedies which usually have been tried, except the last, he has waited for a third case before venturing a decided opinion as to its value. Although he saw these cases only in consultation, especial care was taken to obtain their correct histories. The following is one of his cases: Patient aged 30, third child. Commenced excessive vomiting during the fifth week of pregnancy, which continued, with the usual intermissions, till the tenth week, when we saw her. For several days previous the nausea and vomiting had been almost constant, both day and night. She had become very much emaciated and unable to sit up, even in bed, not having retained any nourishment on the stomach for several days. All the usual remedies had been tried, such as oxalate of cerium, hydrocianic acid, hypodermic injections of morphine, &c., but with little benefit. He suggested the administration by the rectum, morning and evening, of thirty grains of hydrochloral in mucilage, and this to be increased if there was no improvement, or if the specific effect of the medicine was not too decided. An amelioration of the symptoms was obtained by the first injection, and a still more satisfactory one followed the administration of the second. The second day's use of the remedy arrested the vomiting, except at long intervals, and on the third day both nausea and vomiting ceased entirely. There was no return of the symptoms. Some nourishment was taken and retained, even on the second day. From this time the patient rapidly gained strength, and was delivered of a healthy child. Should other opportunities offer for a trial of this plan of treatment, he has decided to commence with larger doses, being convinced that a decided impression, produced by the medicine at first, will require its repetition but two or three times to put an end to the disease, for the time at least. He believes that hydrochloral, administered in this manner, will relieve most cases of nervous or sympathetic vomiting, where there is no inflammation especially. Even in strangulated hernia, on theoretical grounds, it ought to act well, not only in checking the vomiting, but in producing relaxation. He would give it a trial also in cholera.—*Charleston Med. Jour. and Review.*

*Bismuth in Skin Disease.*—To alleviate the intense itching and irritation which accompanies chronic eczema and other forms of skin disease, apply an ointment containing half a drachm of subnitrate of bismuth to an ounce of simple ointment, rubbed up with a little spirits of wine.—*American Medical Weekly.*

*Nux Vomica in Nervous Diseases.*—According to the *Medico-Chirurgical Review,* Dr. de Stefani does not regard nux vomica as an irritant to the spinal cord, but believes that it exerts a depressing action on the ganglyonic system. As this system has numerous relations and sympathies with the cerebro-spinal, nux vomica, acting on both, relaxes the vital tension of the nerves, restores to them their natural conducting power, and also the degree of influence necessary to maintain the harmony of the vital functions of the organs. In acute and serious diseases of the two nervous systems the tolerance of the drug is great; in chronic affections of the ganglionic system it is greater than in that of the cerebro-spinal; and in the organic diseases it is in relation to the gravity of the nervous sympathies. Intolerance of the drug is indicated by stiffness of the lower jaw and of the tongue, and some degree of subsultus in the the lower limbs or in all the body. With reference to the curative action of nux vomica, Dr. de Stefani maintains that it depresses the muscular force if this has been stimulated by hyperasthenia, and stimulates it when it has been apparently depressed by the same cause; that it lowers the pulse when it is hard and vibrating, and raises it when it is small and weak; that it lowers excessive heat of the skin, and warms the skin when it is morbidly cold; that it regulates both the pulse and heat of the skin when they are variable several times in the day; that it relieves ardent thirst; that in costiveness which has resisted repeated purgatives it opens the bowels, and in some cases arrests diarrhœa; that it also arrests spontaneous hemorrhage and relieves hemorrhoids; that it relaxes spasms, removes neuralgic, pleuritic, and rheumatic pains, calms delirium, and removes morbid wakefulness, or awakes patients from morbid sleep, promotes perspiration when deficient or arrests it when profuse, etc., whenever these symptoms are the results of a nervous affection. Nux vomica should not be employed in nervous affections until other remedies have failed. When its use is decided upon, it is necessary to guard against giving too small doses. The dose of the alcoholic extract given by Dr. de Stefani to subjects of middle age suffering from chronic disease is from five to ten centigrammes. In serious cases this may be raised even to thirty centigrammes, combining with it n equal quantity of extract of rhus radicans and some extract of
a enbane.—*Medical and Surgical Reporter.*
h

*Burns and Scalds.*—Dr. Morris, in the *Sanitarian*, thus concludes an article on the treatment of burns and scalds:

"1. Remove the clothing by cutting it from the body.

2. Wrap the patient in blankets.

3. If pain be excessive, administer chloroform, ether, or large doses of opium, and let the necessary dressing be made while the patient is in a state of partial or total insensibility.

4. Produce anæsthesia of the burned or scaled parts by the application of a solution of carbolic acid and morphia. (This solution can be made in almond or olive oil.)

5. After this, wrap the patient in masses of cotton batting.

6. Avoid brandy, and give coffee as a stimulant.

If these simple rules be followed, much suffering may be alleviated, and many a life saved, which otherwise would be lost by the ignorance and mismanagement of attendants."

We regard the above recommendations as good and practical, except, perhaps, the 4th. It would seem that the application of morphine and carbolic acid to an extensive surface might be attended with danger. And we will make this additional suggestion: Let the cotton batting, or, in lieu thereof, some old, soft linen cloths, be freely smeared with the following bland and simple application, the material of which may be found in every household, to-wit: *Albumen of eggs and melted lard*, equal parts, whipped together into a soft, creamy consistence. For many years we have used this simple preparation in burns and scalds, and we have never found anything equal to it. The lard should, of course, be in good condition and contain no salt. It is likewise an excellent application for excoriated surfaces, irritable ulcers and for erysipelas.

R. C. W.

*An Antidote to Chloroform.*—Dr. Schuller has discovered that the nitrite of amyl quickly removes the effects of chloroform on the vessels of the pia mater, and that even in cases of advanced narcotism from the latter drug it rapidly relieves the dyspnœa and labored respiration, restoring the strength of the pulse, and the reflex excitability. This discovery may prove of much practical value where chloroform continues to be the favorite anæsthetic.—*New York Medical Journal, February*, 1875.

*External Use of Tincture of Iron in Erysipelas.*—By Clarence Foster, M. R. C. S.—I wish to direct the attention of my medical brethren to the immense utility of the tincture of iron, locally applied, in arresting erysipelas and many other external diseases when unattended by breach of surface. In simple cutaneous erysipelas, and also in the milder phlegmonous variety, it possesses the decidedly specific effect of subduing, almost at once, the morbid action. I have applied it in numerous instances, and always with the most satisfactory results. So far as my experience goes, it is in these cases incomparably the best external remedy ever used. It seldom happens that more than one painting of the same spot is required; and, having applied it, no other external agent whatever is needed. In scrofulous swellings of the neck its discutient properties are far superior to those of iodine; and where a puerperal breast or inguinal gland in the male has threatened to end in suppuration, the early use of the tincture, every other day or so, with a camel's hair brush, has been sufficient to effect resolution, while in similar cases we find frequently that leeches, poultices and evaporating lotions fail to prevent the formation of matter. Again, this remedy may be applied most advantageously in cases of acute rheumatism, where any particular joint is especially swollen and painful, and also on the inflamed surface surrounding an unhealthy ulcer, or along the course of the absorbents when irritated by a recent, ill-conditioned wound. The well known remedy, ink, as a domestic application in ringworm, has long enjoyed a not altogether undeserved popularity, its ferruginous ingredient. Although the external use of the tincture of iron—first introduced by my father, I believe, some five and twenty years ago—is now pretty common in West Riding, yet its great therapeutic advantages, I have reason to think, are far from being sufficiently appreciated by the profession generally, and I am fully convinced that any surgeon giving the preparation a trial will be amply satisfied with the result.—*Medical Times and Gazette.*

*Diarrhœa Mixture.*—℞. Olei ricini, gtts. xxiv.; sp. chloroform, ʒiss.; sol. morphiæ mur., ℥i.; pul. gum. acaciæ, ʒiiss.; syrupi, ℥iss.; aquæ, ad ʒiv.—M. A dessertspoonful every hour and a half until the bowels are quieted.

*Anomalous Digestion.*—Dr. James Blake writes, in the Pacific *Medical and Surgical Journal:* One of the most striking instances I have seen of anomalous digestion is that of a lady who has what may be called a decidedly weak stomach ; a stomach to which grease in all its culinary forms is nearly poisonous ; which utterly repels against pies and cakes, hot bread and bacon, and, in fact, against most of the articles that are tabooed for weak stomachs; and yet she perfectly digests green cucumbers and green apples. In fact, when suffering from an attack of dyspepsia, and perhaps vomiting even the lightest articles of diet, the first thing that can be digested is a laid green apple, and this, too, late in the evening before retiring. The same patient digests raw turnips and carrots better than when they are cooked. Now, there can be no doubt that there must be something peculiar in the functional action of such a stomach; some peculiarity in the properties of the gastric juice which enables it to act on these uncooked hard masses of woody fibre and cellular tissue in a manner different from the generality of stomachs. Whether this peculiarity in the functions of the organ is connected with anything abnormal in its anatomy, I am unable to say. But there is one fact in connection with this case that might excite a suspicion that it may be so, viz: the existence of what may be called œsophageal dyspepsia, or a habit of retaining a considerable quantity of food in the œsophagus and regurgitating it almost unchanged after it has been down some hours. Whether this is connected with the presence of a diverticulem, or from a simple enlargement of the œsophagus, I am not prepared to say, but in either case we have an approch to the anatomical structure of the stomach found in ruminants, whose normal food is cellular and fibrous vegetable tissue.

*Chloral Suppositories.*—The production of a chloral suppository containing a sufficient proportion of this drug to cause sleep has heretofore been deemed impossible. M. H. Mayet, pharmacien, of Paris, has, however, devised the following formula, by which he manages to get forty-five grains of chloral in each suppository: ℞. Ol. theobromæ, gr. xxx.; cetacei, pulv. chloral., aa. gr. xlv. For one suppository. These suppositories are of good consistence, and may be easily put into use.

*Treatment of Sudden Intestinal Occlusion by Opium.*—M. Antoine Tariote, in *Theses de Paris,* No. 426, 1875, relates two cases which occurred in M. Moutard-Martin's practice, in which appearances of intestinal occlusion, that had shown themselves suddenly, quickly disappeared under the influence of opium given in large doses. The following draught was given : Thebaic extract, ten to fifteen ceutigrammes; white emulsion, 125 grammes; syrup, thirty grammes ; a teaspoonfnl to be taken every hour until symptoms of narcotism appeared. M. Moutard-Martin has given as much as thirty centigrammes of extract of opium in one day. When action of the bowels takes place, the method is discontinued. M. Tariote concludes that intestinal occlusions may be divided into two very distinct categories : 1. Intestinal occlusions of slow origin, caused either by simple accumulation of fecal matters, or by paralysis of the intestine, or diminution of its size in consequence of the presence of foreign bodies, stricture and compression. 2. Intestinal occlusions which make their appearance very abruptly and rapidly, arising from true internal strangulation, invagination, retroversion or twisting of the intestine. In gradual intestinal occlusion, if there be no well-confirmed internal strangulation, opium employed from the commencement, concurrently with applications of ice to the abdomen, or blood-lettings, calms the local irritation and the resultant spasm. It also quiets the accidents arising from the general irritation, anxiety, small pulse, chilliness, etc. This treatment may by itself re-establish the circulation of the gases. The re-establishment of the circulation may be advantageously hastened by the administsation of a purgative.—*London Medical Record,* March 31, 1875.—*Abst. Med. Sciences.*

*Salicylic Acid as a Disinfectant.*—This agent has been recently introduced into the surgical department, and serves a much better purpose than carbolic acid. The great advantage it possesses is, that it is destitute of odor, while it thoroughly deodorizes all discharges that it comes in contact with. It is used in solution directly to the granulating surface by means of a syringe or irrigator. The solution is made by combining and dissolving the following: Salicylic acid, one part ; phosphate of soda, three parts ; water, one hundred parts.—*N. Y. Medical Journal.*

*Puncture of the Bladder for Retention of Urine.*—The following case is reported as showing a very common mode of the primary treatment of urinary retention at the Boston hospital. A boy, fourteen years of age, fell off a pile of boards on the 14th of April, receiving a moderate contusion of the right hip. The next day he had retention, which was relieved after considerable difficulty with a catheter. He had more or less retention till he entered the hospital on the 24th, when it was complete. Repeated attempts had been made to introduce a catheter, but without success, and there was slight hemorrhage from the urethra as a consequence. At this time he was suffering great pain, and the bladder was distended to the umbilicus.

Dr. Ingalls made no attempts at catheterization, but immediately punctured the bladder just above the pubes with the aspirator, and drew off three pints of alkaline urine, with complete relief to the patient. The pain being slight, no ether was used.

The next day the patient was catheterized once. After that he passed his water very well, with the help of an occasional opiate, or warm water enema. He was well on the 29th.

The treatment of retention by aspiration is an admirable one. The bladder is relieved; and the urethra is allowed to rest and recover from the temporary congestion, swelling and tenderness which causes the retention. Generally only one, two or three aspirations are required before the urethra regains its normal condition sufficiently to admit an instrument without difficulty, unless there be a tight, organic stricture. The relief is certain, the pain slight; and the danger nothing, so far as is shown by the pretty large experience of this hospital. The operation may be repeated two or three times a day for several days with safety.—*Med. and Surg. Journal*, Boston.

*Pulsatilla in Amenorrhœa.*—Dr. Pintschovius, of Ketzin, in a letter to the *Allegemeine Med. Centralzeitung*, January 30th, revives pulsatilla as an emmenagogue. He has found it a "sure and convenient article for this purpose." His formula is ℞. Extr. pulsatillæ,; herb. pulsat, aa. q. s.; M. Et div. in pill, 3 grs. each. Sig.—One three times a day. It is a remedy which once enjoyed some reputation in this country.—*Med. & Surg. Reporter*.

*Copabia in Dropsy.*—Several cases illustrating the value of copaiba as a diuretic are given in the *Practitioner*, by Dr. E. L. Dixon. We quote one which was combined with albuminuria: Ellen Gregg, aged 36, married, and has a child eight months old, admitted in Preston Infirmary under my care, 12th May, 1874. There is a loud cystolic bruit at the apex of the heart, the impulse of which has notably increased; pulse very small and irregular. The liver extends two inches below the margin of the ribs, and there is some ascitic fluid within the abdomen. Urine sp. gr. 1.018; albumen $\frac{1}{8}$. No casts were detected under microscope. Considerable œdema of the legs, which has existed five weeks. She has been ill, she says, since Christmas, when she hurt her back; no hemorrhage was, however, produced; has never had acute rheumatism. She was ordered tartrate of iron, with squill, digitalis, etc.; imperial drink, with compound jalap powder occasionally. The urine continued very scanty, and no improvement seemed to take place at all, when, on the 22d, she was ordered to take two copaiba capsules every night. The very next day, the urine of that day being measured the following morning, the amount was nearly trebled, and continued to increase. The albumen diminished, and she was discharged, well, January 16th. The capsules contained from five to ten minims of copaiba each.

*Bicarbonate of Soda in the Treatment of Toothache.*—Dr. Duckworth, of St. Bartholomew Hospital, has related a case of severe toothache which was almost immediately relieved by the use of bicarbonate of soda, after various other remedies had failed. The patient was a boy, suffering from caries of one of the molar teeth. Chloroform was rubbed outside the cheek, and some on cotton wool was placed inside the auditory meatus. These means failing, a plug of cotton saturated with chloroform was inserted in the cavity of the tooth. This was also unsuccessful. Next, pure carbolic acid cotton was placed in the cavity, but no improvement followed, and chloroform was then tried again without success. As a last resource, a solution of bicarbonate of soda, of a strength of about half a drachm to an ounce of water, was tried with the above happy result. He also suggests the use of a warm solution of the bicarbonate, so as to be more sure of the effect of the remedy employed.

*Treatment of Torticollis.*—Dr. Gazzo in *Medical and Surgical Reporter,* remarks: Fifteen years ago I was called to treat an obstinate torticollis affection of the neck, which had resisted the usual general and local means. Five grains of the nitrate of silver were inserted by means of punctures in the subcutaneous tissue of the sternal and clavicular muscles, twice a day, for two days, with marked and satisfactory results. The patient who for three weeks previous had suffered extreme pain upon the slightest motion of the neck, at the expiration of this period was able to move his head with slight incovenience. Friction was ordered, with a stimulating liniment, as follows: ℞. Liniment comp, co., ℥iv; liniment glycerinæ, ℥iij.; tinct. digitalis F., ℥ij.; tinct. aconiti F., ʒij.; M. Ft. liniment to be used two or three times daily, and repeated during five days.

This removed all remaining disease in the affected part.

The next case was one in which no previous treament had been practiced. It was recent; three applications of ten grains of nitrate of silver, upon three succeeding days, so effectually removed the constriction pain, and tenderness, as to allow of the free use of the neck on the fifth day.

*Fucus Poultices.*—A new form of poultice has been introduced to the notice of the profession by M. Lelievre, a Paris chemist, which is proposed as a substitute for linseed meal. It consists of a substitute extracted from the *Fucus crispus,* which can be preserved in sheets like paper. For use, a piece of suitable size is cut and dipped in warm water; it swells rapidly, softens, and can be immediately employed as a poultice. A very favorable report of the substance was presented to the Academy of Medicine by M. Jules Lefort, and MM. Gosselin, Verneuil and Demarquay, who had used it, praised it highly, claiming for it the advantage that the poultices do not dry, do not slip from the place to which they are applied, have no unpleasant odor, do not soil linen, and can be used over and over again many times. The substance would thus appear to have a place intermediate between linseed-meal and spongio-piline, and probably would be capable of much more extensive use than the latter, which is difficult to keep accurately applied, and soon becomes cold.—*Lancet.*

*Hydrastin in Gonorrhœa.*—"As far as internal treatment is concerned, I merely give in the first stage a saline aperient to be continued three times daily for four or five days, together with the following injection: hydrastin, one drachm; solution of morphia (Magendie's) two drachms; acacia mucilage to four ounces: to be used three times daily. This I have employed when inflammation ran very high, without even the slightest ill effects, and have used it in every stage of gonorrhœa with the most beneficial results when every other treatment, both internally and locally, had failed, including red sandal-wood oil. But there is one remark I wish to make regarding the use of injections which medical men generally forget, and that is to tell their patients to micturate previous to its use. Unless this is done, injections in gonorrhœa are useless. Hydrastin is used very much in different parts of the United States and very successfully. My last patient was a farmer, who had had a gleety discharge for seven months. His medical man had quite wearied him out with injections, etc., all to no purpose. I at once tried the hydrastin, and in two weeks he was quite well."—*New Remedies.*

*Diagnosis of Disease of the Liver.*—The *Doctor* says that Prof. Behier lately lectured at the Hotel-Dieu, on the difficulties of diagnosis in certain cases, and an abstract of his observations, by M. Dalbanue, appears in *Le Courier Medical.* He related cases in which cirrhosis of the liver had been believed to exist up to the time of his death, but in which the autopsy revealed abdominal cancer or some other lesion. He says that whenever ascites is the first symptom, and is accompanied by emaciation, an earthy tint, diminution of the area of hepatic dullness, with absence of vomiting, one naturally supposes the symptoms are due to cirrhosis of the liver. On the other hand, if the ascites is preceded by obstinate vomitings which began suddenly, cancer of the stomach may be suspected. As for dilation of the abdominal subcutaneous veins, this appearance is common to both diseases. He concludes that the diagnosis of cirrhosis of the liver is more difficult than is generally supposed, and in three of his cases of cancer the characteristic aspect described by authors was replaced by the earthy tint of cirrhosis of the liver.

*Nitrogenous Diet in Rheumatism.*—It is stated in the *Doctor* that Dr. Andrew, in the new volume of "St. Bartholomew's Hospital Reports," says that he has submitted eight cases of acute rheumatism to nitrogenous diet, which he believes promises better results in this disease than any other treatment. The pains disappeared on the average in 3.75 days, the longest time being 5, the shortest 2. It is to be remarked that only young patients whose powers of nutrition are unbroken are likely to bear with benefit this powerful method of treatment, and even in these it cannot be continued very long. Dr. Parks himself showed that the power of the heart is greatly reduced by this diet within twenty-four hours. It would seem, therefore, that such a plan is only adapted for acute cases.

Nevertheless, Dr. Moss, of the Royal Naval Hospital, Esquimalt, Vancouver's Island, has applied it, though without much success, to *chronic* rheumatism, which, it appears, is very common in that island. The diet adopted was that given by Dr. Parkes, with the addition of tapioca pudding without milk or eggs. It consisted of arrowroot cakes made with butter, and sometimes with sugar or treacles. Dr. Moss gives the details of the weight and quantities. The bowels became regular, the rheumatic pains lessened and dyspepsia disappeared.

*Bromide of Ammonia in Catemenial Excesses.*—Dr. J. K. Black, of Newark, Ohio, has often tested the efficiency of this preparation in non-structural excesses, and he speaks (*Cincinnati Lancet and Observer*, May, 1874,) with confidence of its valuable powers. He says he no more certainly anticipates the arrest of an attack of ague by the administration of quinia than does he anticipate the control of the forms of catamenial excess referred to by the proper administration of the bromide of ammonium. In the administration of the remedy, an essential rule is that its use shall precede the expected period by at least ten days. Its administration only during the crisis will do very little if any good. The sedative influence of the remedy must precede and accompany the stage of ovarian and uterine vascular engorgement, which itself precedes the flow by several days. Any associated disorder, which has even a remote bearing upon the menstrual excess, should, of course, receive appropriate attention.—*American Journal of Medical Sciences*, July, 1874.

*The Treatment of Fractured Patella.*—Prof. Spence, of Edinburgh, remarks (*Practitioner*) that, though the screw hooks designed by M. Malgaigne for transverse fracture of the patella are perfect as a mechanical appliance for maintaining the fragments in accurate apposition, yet they have not been very generally used on account of the pain and irritation of the skin often caused by them. Of late years Mr. Spence has adopted the following method of using these hooks without inserting them through the integument, and it answers its purpose admirably. The two fragments of the patella being held by an assistant in as close apposition as the amount of effusion in the joint will allow, a broad piece of strong mole-skin adhesive plaster is applied close above the upper fragment. This piece should nearly encircle the limb, and, to make it fit around the upper edge of the patella, a crescentic piece is cut out of the centre of the lower margin. A similar piece is applied below the lower fragment. Two or three smaller bits are then placed one over the other, just where the point of the hooks will be fixed, so as more thoroughly to protect the skin. Care must be taken to have the integuments on the stretch when the plasters are applied, and the latter should be put on some hours before the hooks are inserted, so that they may have time to adhere firmly. This may be secured by keeping a bandage firmly applied over the plasters in the interval.

In the same journal, Mr. McGill describes the mode of treatment adopted in these cases by Mr. Teale, of Leeds. He fixes the limb on a straight back splint, or simply places it slightly bent on a pillow between sand-bags, and applies evaporating lotion. He finds that the fragments thus left to themselves, without the use of any hooks, straps or other apparatus, approximate steadily and naturally. The patient is kept in bed for six or eight weeks, and, when allowed to get up, is fitted with a knee-cap of chamois leather. Mr. McGill testifies that the results of this "expectant treatment" are quite equal to those which can be obtained by any other plan. He relates a case in which the distance between the fragments was thus spontaneously reduced from 1¼ inches to ¼ inch in less than five weeks. He gives the following explanation of the process: The separation of the fragments is in the first instance partly due to spasmodic contraction of the quadriceps extensor, which subsides naturally after a few days, and partly to the effusion into the joint which always occurs at the time of the accident; as this is slowly absorbed, the fragments again fall together. Lastly, the fibrous band which is formed between them has, like all other cicatricial tissue, a tendency to contract, and thus completes the approximation.—*London Med. Record.—Abst. Med. Sciences.*

*Ergot in Cystic Paralysis*—Dr. J. Willimsky (*Ceneralblatt for Chirurgie*) made some experiments with ergot upon the bladders of dogs and rabbits, in which he observed that immediate contraction of that organ ensued, and continued for several seconds. He afterward employed it in the form of injections of the fluid extract in two cases of paralysis, with the following results: The first was a marked paralysis occurring in a maiden lady of forty-six; division of a cicatrix between the posterior wall of the bladder and the neck of the uterus proved ineffectual. A small quantity of the fluid extract was then injected daily into the previously cleansed bladder, and a complete cure was effected in fourteen days. The second was a case of acute cystic catarrh, with inability to completely empty the bladder. Three injections at intervals of twenty-four hours afforded entire relief.—*The Clinic.—Med. Examiner.*

*Ingrafting of Tendons.*—M. Tillaux has practiced this operation in a patient who had laceration of the extensor tendons of the ring and little fingers. The operations were done in a month after the accident and when cicatrization was complete. The digital extremities were easily found, but not so the superior extremities. Accordingly the operator incised the tendon of the common extensor, and refreshing the superior ends of the broken tendons of the ring and little fingers, slid them into the place prepared for them, thus grafting them in. The operation was successful, and the patient recovered the use of his fingers.—*Jour. de Med. et de Chir.—Medical Record.*

*Anti-Gastralgic Drops.*—℞. Tinct. nucis vomicæ, tinct. castorei, aa. ℥ss. M. Two drops during the paroxysm, in half a wineglass of infusion of chamomile.

*Digitalis in Typhoid Fever.*—Dr. Bernheim (*British Med. Jour.*) describes his studies of the action of digitalis in typhoid fever. He concludes:

(1) Digitalis when administered in typhoid fever always produces lowering of the temperature. (2) The first effect of digitalis on the temperature shows itself two days after administration; the lowest temperature is obtained on the following day, sometimes only the fourth day after its administration. (3) The lowering is generally progressive, sometimes it terminates by a more rapid fall. In some cases there is a very rapid fall in the interval between two thermometric observations from the evening to the morning or from the morning to the evening. (4) During the action of digitalis the temperature may rise again in the evening, often it is equal or inferior to that of the morning. The greatest remission lasts from a few hours to three days. The temperature then rises. In about two-thirds of the cases it no longer rises to the same height as before the administration of the digitalis. (5) Taking into account the descent of the minimum and the reascent, it may be said that the influence of digitalis on the temperature varies from three to twelve days, the average being seven days. In two-thirds of the cases the thermometer never reaches the original point. (6) The pulse generally falls gradually with the temperature; the temperature rarely falls before the pulse or the pulse before the temperature.

*Ointment for Sycosis.*—Dr. S. Smith, of New London, Conn., sends us the following formula for an ointment, which he has used for several years, with unvarying success, in the cure of this intractable affection: ℞. Acid tannic, gr. xv.; sulphur, gr. xij.; aquæ, ʒ ijss. Apply a quantity the size of a pea to the affected spot every morning and night.

*Treatment of Delirium Tremens.*—Dr. D. H. Kitchen (*Amer. Jour. Insanity*, Jan., 1875), in an elaborate article upon delirium tremens, recommends the following as the most effective treatment: A generous diet is given, full doses of fluid extract of conium during the day to control muscular action, and during the evening hydrate of chloral with tincture of hyoscyamus, repeated until sleep is secured.

*The Remedial Use of Sea Water as a Beverage.*—Dr. Lisle, in the *Buelletin de Therapeutique*, recommends sea-water as often beneficial. He finds that its continued use increases the appetite, facilitates digestion, quickens nutritive changes, and augments the proportion of red corpuscles in the blood. Accordingly he recommends:

1. During convalescence from acute diseases; 2. In the apyretic forms of dyspepsia; 3. In neuroses associated with impoverishment of the blood; 4. In the scrofulous and tuberculous diathesis; 5. In diabetes.

Sea-water may be agreeably administered in bread, in the form of a syrup, or in that of an elixir. Bread made from sea-water can only be procured at the seaside; it is very palatable, and contains nearly five grammes of the mineral constituents of the water in each pound. The syrup is prepared by mixing 250 grammes of sea-water with a sufficiency of sugar and distilled water to make 500 grammes. Each tablespoonful of the syrup contains about twenty-five centigrammes (3¾ grains) of the saline residue of sea-water; from two to five tablespoonfuls may be taken daily.

The formula for the elixir is: sea-water, 200 grammes; rum, 200 grammes; sugar and distilled water, up to 500 grammes. The dose at first is a tablespoonful three times a day.

To the obvious objection that a pharmaceutical mixture of the saline constituents of sea-water in their due proportions would serve the same remedial purposes as the sea-water itself, Lisle replies that the efficacy of all natural mineral waters is very much greater than that of the manufactured counterparts, the testimony of those who have instituted comparative trials being all but unanimous on this point.

*Diphtheria.*—Dr. J. Lewis Smith (*N. Y. Medical Record*) says: The local treatment consists in the application to the fauces, every three hours, of the following, which has given me better satisfaction than any other medicine for local treatment which I have employed: ℞. Acidi carbolici, gtts. v.; liq. ferri subsulphat, ʒ ij.; glycerin, ℥ i. M. The brush was employed in preference to the probang, as it is less painful, and where there is a pseudo-membrane does not produce hemorrhage by the detachment.

*Iodoform in Chronic Suppuration of the Middle Ear.*—Dr. Frank H. Rankin, Assistant Surgeon to the Manhattan Eye and Ear Hospital, contributes to the *Mew York Medical Journal* (May, 1875) an interesting article on this subject.

Until within a very recent period the use of iodoform was confined almost exclusively to venereal troubles, and chiefly employed as a topical remedy. Its domain of usefulness however, was not allowed to remain contracted to so narrow a field, but is now constantly widening, and every year is showing the importance of this very useful drug.

Though containing ninety-six per cent. of pure iodine, it is totally devoid of corrosive properties, and has not the slightest local irritating action. It can be applied to highly-inflamed and sensitive tissues, without exciting pain; in fact, it acts as a local anæsthetic. Besides possessing the stimulating effect of the ordinary iodine and iodides, it acts as a local tonic, and possesses highly disinfectant properties. We would naturally conclude that a drug possessing so many valuable properties would be of decisive benefit in chronic suppuration of the middle ear, and the highly favorable result obtained in the cases just given justifies us in asserting that in iodoform, properly used, we have a very important agent in checking the chronic discharges from the tympanic cavity.

Before using the iodoform, which should be in a finely powdered state, it is, of course, essential to see that all secretion shall have been *thoroughly* removed from the middle ear, and the parts well dried with cotton on a holder. The instrument I have used for blowing in the iodoform is an insufflator, similar to that used for the throat, with only this difference: that the tube, instead of being curved, is straight.

*The Cyanides in Acute Articular Rheumatism.*—M. Luton (*Bulletin de Therapeutique*) strongly recommends the use of the cyanides in the treatment of acute articular rheumatism. The cyanide of zinc he gives in average doses of one and one-half grains daily, the cyanide of potassium in rather smaller doses. As a total result from many observations, M. Luton states that it is certain that cyanides cure acute articular rheumatism in its fundamental form and its diverse transformations.

*The Sulphuret of Carbon in Chronic and Atonic Ulcers.*—M. Guillaumet writes (*Journal de Therapeutique*) in warm terms on the value of this substance, first introduced by M. Michel in 1867, and since abundantly tried in Dr. Costilhe's wards at the St. Lazare, in cases in which all other remedies have failed. Owing to its nauseous odor, it is applied as rapidly as possible over the surface of the wound, by means of a morsel of charpie, the surface being then covered over with a fine powder of nitrate of bismuth or starch. In recent ulcerations, one or two applications suffice, but five or six applications may be required in old ulcers before any appreciable modification is obtained; but then cicatrization advances rapidly. The following are the conclusions arrived at: 1. Sulphuret of carbon is a very powerful cicatrizer; 2. Its action is rapid and quite local, not producing any of the accidents which attend the prolonged inhalation of its vapors; 3. Its application is accompanied by pain, which is sometimes sharp, proportioned to the susceptibility of the patient, but in most patients of very short duration; it is immediately followed by a period of anæsthesia, which, however, is not constantly present. When it exists it lasts for several hours, while the painful period does not last more than from twenty to sixty seconds; 4. The sulphuret acts upon wounds of different origin and nature (syphilitic, scrofulous, diphtheritic, etc.), and modifies all of them advantageously; 5. It is a valuable agent in the treatment of all wounds and ulcers which possess the common characters of chronicity and atony.—*Med. Times and Gaz.*

*Guarana in Chronic Rheumatism.*—Dr. E. A. Rawson (*Irish Hospital Gazette*) strongly recommends the use of guarana for the pains of chronic rheumatism. When the pain comes on with sharp stings, guarana acts like magic. When it is dull in character the drug is slower in action, several doses being needed to produce decided effects. Gravelle, writing in 1840, says that guarana stimulates and at the same time soothes the gastric system of nerves, and reduces the excited sensibility of the cœliac plexus, thereby diminishing febrile action and strengthening the stomach and intestines, particularly restraining any excessive mucous discharges, at the same time increasing the action of the heart and arteries, and promoting diaphoresis.

*On the Use of Chocolate in Chronic Intestinal Catarrh.*—A series of articles from the pen of Dr. Karner has lately appeared in the *Allgemein Weiner Med. Zeitung*, in which he shows the value of this substance as an article of food. He refers especially to its use in chronic intestinal catarrh, and cites, among others, the following typical case of chronic catarrh of the intestines to illustrate its action in the simplest manner: "Rosalia M., aged seventeen months, poorly developed and nourished, suffered from intense meteorism, numerous thin, fluid, feculent discharges, which alternated from time to time with normal stools. There was considerable emaciation, and the child also had intertrigo, of which there were frequent relapses. The diarrhœas could be attributed only to poor nourishment. After strictly regulating the diet, small doses of Dover's powder and acetate of lead were first administered, and in three days were substituted by the chocolate, of which a cupful was given daily. A dessertspoonful of the powder sufficed for a cup of chocolate. The mother was also instructed to allow the child as little fluids to drink as possible. The result was astonishing. The discharges decreased in number day by day, the weight of the child rapidly increased, and after a few weeks it had perfectly recovered; a remarkable change for the better was observed in its bodily development, and the intertrigo had not recurred."

Such cases of chronic intestinal catarrh admit of no experimentation, as the physician is usually called at a time when the symptoms of disease have already assumed threatening aspect. "By this diarrhœa," says Niemeyer, "the previously healthy, well-nourished child is at first but little affected; but some fatal judgment often asserts it to be a safety-valve that protects the child from convulsions during teething, and that must not be stopped. Hence it often happens that the doctor is not called till the child has become flabby and relaxed, and then it is frequently difficult to master the disease; the diarrhœa continues, the child emaciates more and more, and a large number of children die during their second year from chronic catarrh of the intestines."—*London Med. Record.*

*Wood-Sorrel in Epithelioma.*—The dried extract of wood-sorrel has been used as a dressing in epithelioma, and found to be more serviceable than anything else in relieving the pain.

*Treatment of Chorea by Arsenic in Large Doses.*—By Eustace Smith, M.D.—Arsenic has long been regarded as an useful therapeutic agent in the treatment of chorea, but it may not be generally known that the curative value of the drug is greatly increased by administering it in full doses. The tolerance of children for arsenic is a matter of common observation, and this tolerance is especially marked in the case of a non-febrile disease, such as chorea, where there is no increased irritability of the digestive organs. To a child between the ages of five or six and twelve, the subject of this complaint, Fowler's solution may be given in doses of ten minims three times a day, directly after the meals. The influence of this treatment upon the disorder is seen almost immediately, and it is rare for any of the physiological effects of the drug to be observed. By this means, cases of the disease which had resisted smaller doses of arsenic, may be cured in a few days, and even severe cases seldom last longer than a fortnight or three weeks.—*British Medical Journal.*

*Salicylic Acid in Extensive Burn.*—There has been in hospital for many months a case of extensive burn, in which different applications have been tried. Every new dressing succeeded well for a time, but soon it ceased to prove of advantage. The last agent that has been used, and is used at present, is salicylic acid. The effect is more beneficial than that obtained by any of the former remedies. The method of using it is to form an emulsion with olive oil, one part of the salicylic acid to sixteen parts of oil. This mixture is painted over the ulcerated surface once or twice a day. It gives rise to a slight smarting pain when first applied, but that soon passes off.—*N. Y. Medical Journal.—Bellevue Hospital.*

*Bromide of Potassium in Obstinate Vomiting.*—Foreign physicians, Drs. Gerabetti and Laborde, have obtained excellent results from enemeta of bromide of potassium, in doses of one-half to two drachms, in cases of inordinate vomiting during pregnancy, and in diseases of the other abdominal viscera.—*Lancet.*

J. B. M.

*Magendie's Solution of Morphia.*—Take of sulphate of morphia, 16 grs.; distilled water, 1 fluid ℥.; sulphuric acid, 4 drops. Make a sulution.—*Druggists' Circular.*

*Solubility of Salicylic Acid.*—Salicylic acid is very sparingly soluble in cold water, requiring at least five hundred times its weight of that fluid to hold it in permanent solution. The addition of certain alkaline salts increases its apparent solubility. Among these sodium phosphate has been especially recommended. It seems probable that an acid phosphate and a salicylate of sodium are formed, as the solubility of the sodium phosphate itself is also greatly increased. If this is the true explanation, it is probable that the antiseptic powers of the salicylic acid are partially lost in the solution made of the acid by this salt. Ammonium phosphate has also been employed, but is probably liable to the same objection. Sulphite of sodium is now recommended as yielding a solution possessing antiseptic powers of a high order. One part of salicylic acid, two of sodium sulphite, and fifty of water, furnish a solution free from irritating properties, and especially to the treatment of zymotic diseases.

Glycerin warmed will dissolve one-fiftieth of its weight of salicylic acid, and the solution may then be diluted with water to any desirable extent.—*Detroit Review of Medicine.*

*Obstinate Vomiting in Pregnancy Treated by Dilatation of the Os Uteri.*—Dr. E. Copeman (*British Medical Journal*) reports three cases of vomiting during pregnancy, which, after all other known means had failed, were completely relieved by dilating the os uteri with the forefinger. In the first case he was endeavoring to bring on a miscarriage, to prevent death of the woman from exhaustion. Having failed with the means at his disposal to do more than dilate the os, he desisted. While waiting for a better instrument the vomiting ceased and never recurred—the woman going to the full period of pregnancy—was then delivered of a healthy child, and made a good recovery.

*Characteristically Spanish.*—In a lecture on the relative procreativeness of the sexes, Dr. Schlemmer (*Allg. Wiener Med. Zeitung*) mentions the fact that Isabella of Aragon, about the latter part of the fifteenth century, issued a decree fixing (not limiting) the number of conjugal embraces per day at six. What an infliction! The men must have been equal to goats in the fifteenth century.—*Medical Times.*

*Retained Placenta.*—In reference to some cases of retained placenta that had been treated by forcible removal, which he regards as a dangerous practice, Dr. Lineard, of Caen, calls attention to the fact that many years ago he published a simple procedure, which he has always found as effectual as it is safe and easy, and which is also a very efficacious means for the prevention of after-pains and uterine hemorrhage. It consists in the injection of the umbilical vein with cold water. A clean section should first be made, so as to bring the vessel plainly into view, and also to shorten the cord, which should not be more than from twenty to thirty centimetres in length. A syringe, containing at least 150 grammes, and having a long fixed canula, should be employed. The colder water used, the less is the quantity that need be injected ; so that while 150 grammes suffice at the ordinary temperature of winter, twice or thrice as much may be required in summer.—*Gaz. Des. Hop.*

*Quinia in Parturition.*—Dr. O. B. Stafford, of New Boston, Illinois, claims for quinine notably beneficial properties in accelerating parturition. He cites several cases of apparent uterine inertia, in which the administration of seven to ten grains was followed, in from twenty to thirty minutes, by strong expulsive efforts, and speedy termination of the labor. Dr. S. states that it does not cause the *spasmodic* uterine action, which, in his hands, often follows ergot, and the risk of hemorrhage is lessened, the uterus contracting firmly and expelling the entire placental mass with but little difficulty.

We should like to learn the experience of others on this interesting subject. J. B. M.

*Salicylic Acid.*—In chronic cystitis, the bladder has been washed out with a solution containing one part in five hundred of water. The method of washing out the bladder has been to make four injections, of one ounce each, every morning and every evening. The acid not only removed the disagreeable odor of the urine, but in a short space of time freed it from pus. In empyema a solution of the same strength has been employed with very valuable results. It is used under the same circumstances as carbolic acid was formerly. In dressing suppurating surfaces, it appears to have a stimulant effect on the granulations, somewhat similar to that of carbolic acid.

*New Method of Administering Raw Meat.*—The following facts respecting M. Yvon's process are from *Brit. Med. Journal.*

He takes raw beef steak, 250 parts; blanched sweet almonds, 75 parts; bitter almonds, 5 parts; white sugar, 80 parts. The blanched almonds are pounded with the meat and sugar in a marble mortar so as to obtain a homogeneous paste. When reduced to a pulp this paste is of a pale pink color and has a very agreeable flavor, not in the least like raw meat. If placed in a cool dry place it will keep for some time. If it be desired to give it in a liquid form it may be diluted with water.

An emulsion may be prepared as follows: Raw meat, 50 parts; blanched sweet almonds, 15 parts; bitter almonds, 1 part; white sugar 16 parts; are all pounded in a mortar, as in first formula; the quantity of water needed is gradually added, and the whole mixture passed through a sieve. The addition of some yolk of eggs renders this emulsion more nourishing.—*Detroit Review.*

*Chorea.—Treatment by Anstie.*—The late Dr. Anstie, in the *Practitioner*,, June, 1874, relates the following experience concerning the treatment of chorea:

The spraying of the skin the entire length of the spine was found very successful in several cases. When combined with the internal use of arsenic, more favorable results were obtained than by any other remedy. In debilitated subjects, cod liver oil and zinc were useful. Zinc is unreliable, and chloral viewed with distrust. In the milder and more chronic cases, where the nervous system is not too much disorganized and protracted, the shower-bath is worthy of being called a sheet anchor.

*Strychnia as an Anti-Emetic.*—℞. Sig.; aquæs trych. m. xx ℥iv; M. One drachm every two hours. Dr. Thos. E. Dupreis reports, in the *Canada Lancet*, a case of vomiting so excessive and uncontrollable as to produce alarming prostration, arrested, entirely, in a few hours, by the use of the above prescription.

J. B. M.

*Incontinence of Urine.*—If they blistered the child's sacrum, put it on tinct. of iron and belladonna, and gave no salt in its food, they would have very few cases of incontinence of urine.—T. M. MADDEN, *Dublin Journal Medical Science.*

*Russian Cure for Drunkenness.*—By H. Haurowiz.—For some time past *Herba serpylli* (wild thyme) has been used with great success to effect a permanent cure of drunkenness; in case of a relapse (only after years), a short treatment will effect a cure again. The treatment consists in making an infusion of wild thyme (1½ oz. to 1½ pints), and give the patient a teacupful every half hour. The next day it is given every two hours, and then 4 to 6 times a day until the cure is complete, which generally takes from two to three weeks. The effects are in the following order: vomiting, diarrhœa, increased urine, strong transpiration; then, generally, increased appetite and craving for acidulous beverages. Diet: easily digested food, and lemonade or other acidulous liquids.—*American Journal of Pharmacy.*

*Ergotin in Fibroid of the Uterus.*—Dr. Bartholow, in the *Clinic*, had recently under his care a case of submucous fibroid of the uterus, for the cure of which he resorted to the hypodermic injection of an aqueous solution of ergotin in one-grain doses night and morning. On the afternoon following the injection the patient began to feel expulsive pains, which continued to increase in severity. After two weeks the injections were practiced only once daily.

One week later the pains had diminished in severity, and on examination, the neck of the uterus was found very much dilated and plugged with a tumor, which was then easily removed by the écraseur. The patient fully recovered, and has suffered none since the removal.—*Medical Times.*

*Sciatica.*—The treatment of sciatica is based on the view that it is usually due to malaria or syphilis, and for this purpose quinia is first given to the extent of sixty grains in twenty-four hours, followed the succeeding day by thirty or forty grains. If this fails to benefit, anti-syphilitic treatment usually proves effectual.

*A Convenient Local Anodyne.*—Cake of elastic collodion, one ounce; hydrochlorate of morphia, fifteen grains. Dissolve the morphine salt in the collodion. Spread with a camel-hair brush some of this solution on the painful part, and place some oiled silk over the spot. The effect is stated to be most satisfactory.—*London Lancet.*

